# Psychophysiological stress response after a 6-week Mindful Self-Compassion training in psychiatric rehabilitation inpatients: a randomized post-test only study

**DOI:** 10.3389/fpsyt.2023.1098122

**Published:** 2023-07-18

**Authors:** Andrea Andorfer, Sabina Kraler, Paul Kaufmann, Ewald Pollheimer, Christoph Spah, Jürgen Fuchshuber, Christian Rominger, Claudia Traunmüller, Andreas Schwerdtfeger, Human-Friedrich Unterrainer

**Affiliations:** ^1^Department of Psychiatry and Psychotherapeutic Medicine, Medical University of Graz, Graz, Austria; ^2^CIAR: Center for Integrative Addiction Research, Grüner Kreis Society, Vienna, Austria; ^3^Institute of Psychology, University of Graz, Graz, Austria; ^4^Center for Psychosocial Health, Sonnenpark Neusiedlersee, Rust, Austria; ^5^Department of Psychoanalysis and Psychotherapy, Medical University of Vienna, Vienna, Austria; ^6^Department of Religious Studies, University of Vienna, Vienna, Austria; ^7^Faculty of Psychotherapy Science, Sigmund Freud University, Vienna, Austria

**Keywords:** heart rate variability, blood pressure, mindfulness, self-compassion, stress task, clinical trial

## Abstract

**Objectives:**

Mindfulness-based interventions (including self-compassion interventions) are effective in improving stress management at psychological and physical levels. Mindful Self-Compassion (MSC) is a newly developed program particularly aimed at increasing self-compassion. The main objective of this study was to determine whether the psychophysiological stress response during a social-evaluative speaking task differs in inpatients participating in the MSC or the Progressive Muscle Relaxation (PMR) program at the end of their 6-week psychiatric rehabilitation stay (i.e., post-test only design).

**Method:**

Data from 50 inpatients (25 MSC, 25 PMR, 35 female) aged 19 to 76 years (*M* = 47.22, *SD* = 12.44) were analyzed in terms of psychophysiological stress response. For this purpose, heart rate variability, heart rate, and blood pressure were assessed together with several psychometric variables: positive and negative affect (PANAS), subjective stress perception (Visual Analog Scale), self-compassion (Self-Compassion Scale), cognitive reappraisal and suppression (Emotion Regulation Questionnaire), psychological distress (Brief Symptom Inventory-18), and appraisal and rumination (selected items).

**Results:**

After correction for alpha inflation no differences in the psychophysiological stress response and psychometric parameters between the MSC and PMR group were found.

**Discussion:**

In general, our results indicate that MSC is not superior to PMR training. However, more research with clinical randomized controlled trials investigating larger samples are needed to further affirm these initial findings.

## Introduction

Stress plays an important role in psychiatric diseases and can be responsible for the frequency and actual course of mental illness (1, 2). According to Selye (3) stress is related to a physical reaction in the body. It helps us to survive by converting the unbalanced state in our body to homeostasis (4). Immediate adaptations include a rise in Heart Rate (HR), Blood Pressure (BP), and glucose consumption, concurrent lack of appetite, activation of the immune system and mobilization for energy regulated by the Autonomic Nervous System (ANS) comprising the Sympathetic Nervous System (SNS) and the Parasympathetic Nervous System (PNS) (5–7). Parasympathetic activity is associated with vagal tone which can be measured by the changes in duration of heartbeats (8). These rapid beat-to-beat changes are predominantly due to the PNS, which is reflected in Heart Rate Variability (HRV) and can be used to assess ANS function (9, 10). Accordingly, the brain and heart are bidirectionally connected, as the brain influences the heart and vice versa (10, 11).

### The role of HRV for stress coping

Elevated HRV, which refers to greater variability and higher vagal tone, signifies greater stress adaptability (12). Additionally, it has been associated with more positive emotions, more enjoyment of social interaction, less negative emotions during stressful tasks and higher social connectedness (13–15). In contrast, lowered HRV has been suggested to be a marker for all types of diseases and can predict mortality (16, 17). In correspondence to this, various mental diseases such as panic disorder, posttraumatic stress disorder and depression are linked with an imbalance in the ANS, as evidenced by attenuated HRV compared to healthy controls (18–20). The Neurovisceral Integration Model, introduced by Thayer and Lane (21), offers a possible explanation regarding the relationship between psychopathology, stress, and HRV. It postulates that stress could impact brain function, thus hampering adaptive and flexible behavior as indicated by low HRV (10, 22, 23). Therefore, developing effective treatment programs to counter stress associated with psychopathology is of special interest in scientific research (24).

### Mindfulness-based interventions (MBI) in psychiatric diseases

Treatment methods of high relevance and increased recognition in recent years are MBI (25, 26). These techniques are about accepting the reality we live in, letting go off thoughts and living in the here and now, which could facilitate psychological well-being (27). Evidence showed efficacy of MBI in reducing stress, anxiety, and depression in clinical populations (28–30) as well as in healthy subjects (31–33). Furthermore, they appeared to be linked with a substantial increase in self-compassion, quality of life, coping with problems, happiness, resilience and overall psychological well-being (34–37).

### Mindful Self-Compassion (MSC)

A specific MBI essential in the present study is the MSC program founded by Neff and Germer (38). Self-compassion has gained increased attention since its introduction in 2003 (39) and can be described as “the ability to be kind and understanding toward ourselves when we suffer, fail, or feel inadequate” (40, p. 861). The program contains the three important aspects: self-kindness versus self-judgment, mindfulness versus over-identification, and common humanity versus isolation (40). MSC comprises an 8-week program, with one session each week (2.5 h) and a half-day silent meditation retreat (38). It consists of guided sessions including various topics regarding self-compassion (i.e., practicing loving-kindness) and additional homework which enables to practice self-compassion in a formal (sitting meditation) as well as an informal (during everyday life) way (38, 40). Neff and Germer (38) demonstrated that the MSC program improved self-compassion, mindfulness, sympathy for others, life happiness, as well as well-being, and lowers stress, anxiety, and depression.

Self-compassion has shown to be repeatedly associated with well-being and coping with unpleasant or stressful life events (41–46). People high in self-compassion showed decreased worry, rumination, subjective stress, and positive emotion regulation (47–49). In sum, self-compassion is a well-established program to reduce psychological stress and help to better cope with stressful situations (50–52). However, to the authors’ knowledge, there are few findings on self-compassion and physiological stress reactivity. In general, research demonstrated that mindfulness could buffer physiological stress responses (53, 54). Correspondingly, literature showed an increase in HRV and decrease in BP during mindfulness training (55–57). Additionally, self-compassion and mindfulness training could lead to better stress coping and buffer physiological stress (58–61). However, findings are heterogenous (62–64).

### The present study

In the psychiatric rehabilitation clinic Sonnenpark Neusiedlersee in Rust, Austria, the MSC program was modified to fit the typical 6-week length of stay at the clinic to see if this form of MSC shows positive results. While some of the meditations and exercises, such as the inquiry, are identical to the 8-week MSC course, other parts are modified to suit the patients need in the rehabilitation clinic. Gaiswinkler et al. (51) demonstrated higher self-compassion and happiness after 6-week MSC program in comparison to the active control intervention of Progressive Muscle Relaxation (PMR) in this rehabilitation clinic. Moreover, they observed psychiatric and quality of life parameters improving in both groups to the same extent (51). PMR is a very well-established and empirically strong validated relaxation technique (65). Individual muscle groups are tensed and loosened immediately after to increase inner relaxation (66). More specifically it can be useful to decrease stress (67). Since it does not include the mindful/ self-compassion aspect it previously has been chosen as an adequate option for an active control group (68, 69).

### Research aims

The primary aim of this study was to investigate if rehabilitation in-patients who received MSC training show less pronounced stress reactions compared to patients who received PMR training regarding psychophysiological stress reactivity using a social-evaluative stress paradigm. HRV, HR, BP, Positive and Negative Affect (PA/NA), and Subjective Stress Perception (SSP) were compared between groups. So far, the MSC program has only been explored on a psychometric level (51). Therefore, this study served to explore the MSC program on a biometric level in a clinical setting. We expected lower physiological responses to the stressor in the MSC group (i.e., lower HRV decrease, HR increase, and blood pressure increase, from baseline to stress). For secondary outcome, we intended to investigate whether Self-Compassion, Emotion Regulation (Cognitive Reappraisal and Suppression), and Appraisal are higher in the MSC compared to the PMR group. Furthermore, we expected that Rumination and Psychological Distress should be higher in the PMR group.

## Method

### Participants

The presented study is part of a broader study (*n* = 170) which has not yet been published. Within that study, patients were randomly assigned into two groups (MSC, PMR) prior to their start of the rehabilitation stay. For the present study, the randomized allocation was adopted. Participants were adults attending a 6-week psychiatric rehabilitation stay at Sonnenpark Neusiedlersee clinic, located in Burgenland, Austria. All exclusion criteria of the rehabilitation clinic applied to the current study, such as acute suicidal and psychotic episodes or acute addiction disorders, determined by the treating psychiatrist at the clinic. Besides, people with severe cardiovascular diseases were not included. In this investigation, 59 participants were recruited of which nine were excluded (4 = drop out, 3 = no speech, 1 = cardiac arrythmia, 1 = previous stroke). All participants signed a consent form and the investigator ensured that participation was voluntary and withdrawal from consent throughout possible. After the examination, they got a coffee voucher from the in-house café. A sample of 50 individuals (35 female) aged from 19 to 76 (*M* = 47.22, *SD* = 12.44) was examined (25 MSC, 25 PMR). The study was ethically approved by the ethics committee of the University of Graz (GZ. 39/84/63 ex 2020/21).

### Measures

#### Physiological assessment

A mobile electrocardiogram [ECG; VarioPort (70)] provided by the University of Graz with sampling rate of 256 Hz was used to measure HRV and HR non-invasively (Becker Meditec, Karlsruhe, Germany). Three electrodes were placed on the right collar bone, below the left ribcage and on the lower abdomen [modified Einthoven Lead II; (71, 72)]. As indices of HRV established time-domain (Root Mean Square of Successive Differences (RMSSD); Standard Deviation of NN Intervals (SDNN)) and frequency-domain measures (High-Frequency HRV (HF-HRV [0.15–0.4 Hz]); Low-Frequency HRV (LF-HRV [0.04–0.15 Hz])) were assessed (73, 74). RMSSD is mostly used in HRV research and indicates mainly vagal, parasympathetic activity as well as HF (10, 75). SDNN and LF represent the cumulative variance of sympathetic and parasympathetic function (74, 76). Respiration rate, assessed via a respiration belt, was added as a control variable (73). An automated BP device (Bosch + Sohn, Boso Medicus (77),) was used to assess Systolic (SYS) and Diastolic (DIA) BP, and was applied on the right upper arm.

#### Treatment intervention

**Figure 1:**
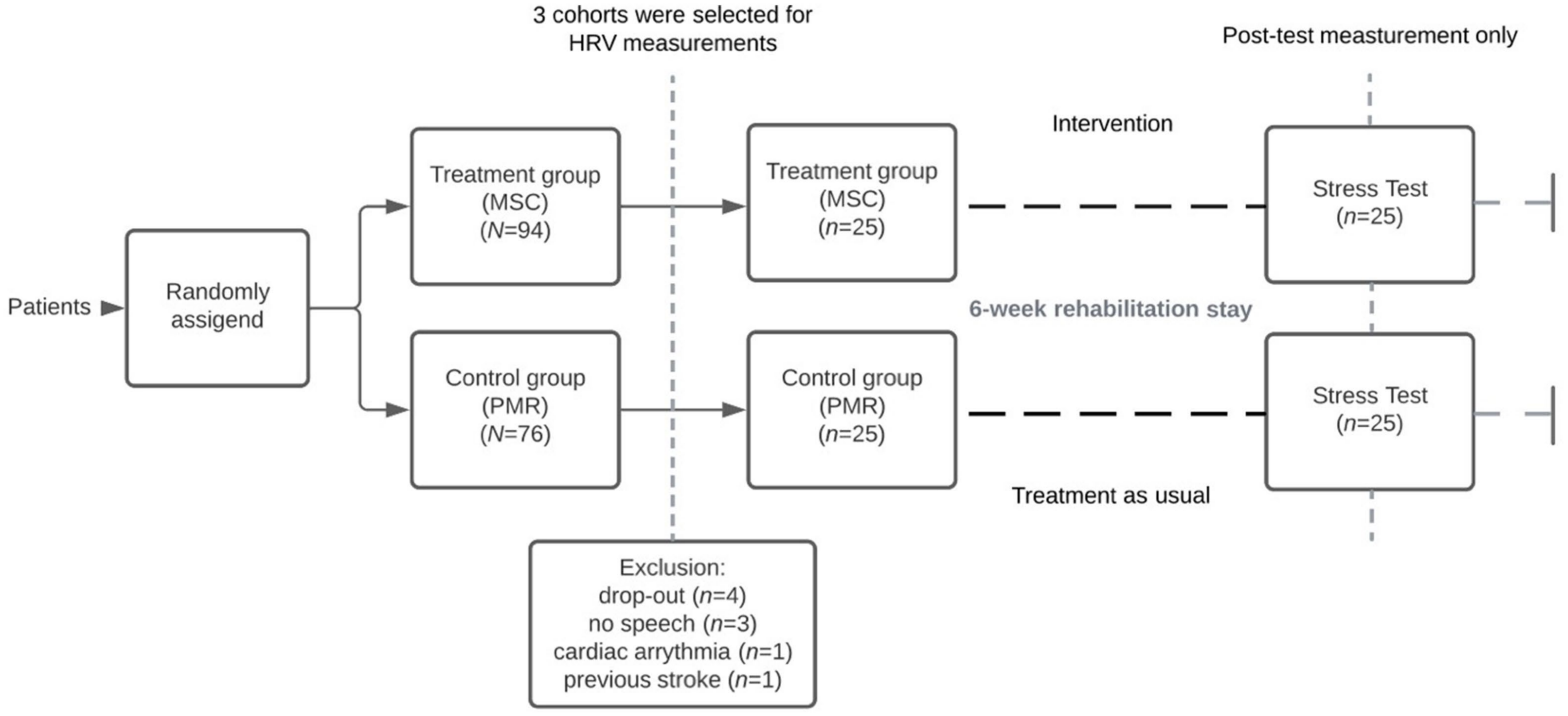
Posttest-only control group design.

Before the arrival in the rehabilitation clinic an independent work counsellor assigned patients randomly to the MSC and PMR group with permuted block for gender, age, and psychiatric diagnosis, which allowed maintaining a balance between treatment groups (78). Both interventions took place once a week for 75 min over 6 weeks and were guided by an experienced MSC or PMR trainer. On the basis of this initial assignment, the patients also participated in the present experimental study (see Figure 1).

#### Stress induction

The stress intervention was based on common social-evaluative stress protocols that have proven to be valid in inducing stress comparable to a negative stress situation, such as the Trier Social Stress Test (TSST) or public speaking tasks (79–85). Participants were prompted to give a 5-min speech introducing themselves for a job offer of their choice. They were filmed and told that the speech was going to be evaluated by experts. Beforehand, they could prepare some ideas by means of questions. After 3 min the judge stopped the speech. If the participants finished their speech before the expiration of time, the investigator asked standardized questions to not stop the flow of speech.

#### Psychometric assessment

For primary outcome: The Positive and Negative Affect Schedule [PANAS; (86)] measures PA and NA with 10 adjectives each on a 5-point Likert scale (1 = *not at all* to 5 = *extremely*). Participants rated how much the adjectives described their emotional state at that moment. Internal consistency (Cronbach’s α) was high (PA: baseline = 0.86, speech prep. = 0.92, recovery = 0.93; NA: baseline = 0.87, speech prep. = 0.86, recovery = 0.87).

Participants’ SSP was assessed on a visual analogue scale (0–100) where 100 (*extremely stressed*) was the highest degree to which they perceived the situation as stressful (81, 87).

Appraisal items were administered with a 6-point Likert scale (1 = *not at all* to 6 = *extremely*). A Demand Resource Evaluation Score (DRES) was formed by subtracting means of evaluated demands (task demand/threat) from means of resources (coping, performance, perceived control, experience) (85, 88, 89). A positive score stands for an Appraisal more of a challenge state and a negative score for threat state (88). Internal consistency was acceptable (threat = 0.78, resources = 0.83).

Rumination was assessed with seven items based on the Rumination Thought Style Scale (RTS), the Rumination-Suppression Scale (RS-8), and the Thoughts Questionnaire [TQ; (90–92)]. Participants rated on a 6-point Likert scale (1 = *strongly disagree* to 6 = *strongly agree*). Cronbach’s α was 0.83.

Secondary outcomes included the Self-Compassion Scale [SCS-D; (93, 94)] which assesses Self-Compassion via 26 items on a 5-point Likert scale (1 = *almost never* to 5 = *almost always*). Cronbach’s α was observed to be 0.89.

The Emotion Regulation Questionnaire [ERQ; (95)] tests Reappraisal and Suppression as emotion regulation strategies using 10 items on a 7-point Likert scale (1 = *strongly disagree* to 7 = *strongly agree*; Cronbach’s α_reappraisal_ = 0.84; Cronbach’s α_suppression_ = 0.77).

The Brief-Symptom Inventory [BSI-18; (96)] measures the Global Severity Index (GSI) of psychological distress via 18 items on a 5-point Likert scale (0 = *not at all* to 4 = *extremely*). Cronbach’s α was 0.88.

### Procedure

Participants were tested in the last week of their stay at the clinic (post-test only experimental design; see Figure 1). They were requested not to consume caffeine, alcohol, nicotine, and sugary drinks, as well as to refrain from physical exertion 2 h before the testing (73, 97, 98). Initially, participants’ height, hip, and waist circumference were measured, and anamnestic data collected. During the recording, participants sat quietly in an upright position and were asked not to move (98). HRV and HR were measured continuously. BP was measured 6 times (baseline & recovery: min 2:30, 4:00; speech prep.: min 0:30, 2:00). For the 5-min baseline recording, landscape pictures were presented on a screen. After the 3-min speech preparation, the actual 3-min speech took place. The recovery phase followed, identical to baseline. After baseline, speech preparation, and recovery PANAS and SSP were presented. Appraisal was collected after speech preparation and recovery and Rumination after recovery. Finally, participants were debriefed. It was made clear that the video recordings were not evaluated at all and were deleted immediately, but only served to reinforce stress (99). One female judge (S.K.) was present during all stress induction, wearing a white coat. There was always the same judge (S.K.). One camera was mounted right beside the computer screen. SCS-D, ERQ, and BSI-18 were presented on the last day of the 6-week stay. The time of day when the stress induction took place could not be controlled, otherwise it would not have been possible to give all patients an appointment for testing.

### Data parametrization

HRV analyzes were conducted with the software packages AcqKnowledge 4.3 (100) and Kubios HRV Premium 3.3.1 (101). R-R time series (interbeat intervals) were interpolated with 4 Hz (101). Fast Fourier Transformation (FFT) was applied to quantify HF-HRV [0.15–0.4 Hz] and LF-HRV [0.04–0.15 Hz] with 180 s window and no overlap (73, 102). The smoothness priors algorithm was used for detrending (101). To control for artifacts, HRV data were visually inspected by the examiner and corrected with the automatic correction algorithm in Kubios software if necessary (101). For further analyzes, only data containing less than 5% of artifacts were considered. An exception was made for one subject due to the small violation of the limit during recovery (artifacts = 5.16%). One person had to be excluded due to excessive artifacts (baseline = 23.63%, speech prep. = 20.09%, recovery = 25.85%). In addition, one participant could not be included in HRV, and HR analyzes due to flawed recordings. RMSSD, HF, SDNN, LF, and HR means of the last 3 min of the 5 min recordings (baseline, recovery) as well as means of the 3 min recording (speech prep.) were analyzed which seemed a sufficient length for ultra-short term HRV measures (74). Due to the sensitivity to movement in HRV recordings, an *a priori* decision was made to incorporate speech preparation as a stress phase (73). Research shows, stress anticipation can also trigger adequate stress responses (103). Prior to analyses, RMSSD and SDNN data were subjected to a natural log transformation to account for skewness (73, 104).

### Statistical analyzes

Statistical analyzes were done with SPSS, version 27 by IBM. Group comparisons regarding anamnestic variables were conducted by means of chi-square and unpaired *t*-tests. For primary outcome, separate two-way mixed ANOVAs with the between-subject factor group (MSC, PMR) and the within-subject factor time (baseline, speech prep., recovery) regarding psychophysiological measures (HRV, HR, BP, PA, NA, SSP) were performed. For post-hoc analyzes, Bonferroni pairwise comparisons were implemented. Secondary outcome analyzes included unpaired *t*-tests with the independent variable group (MSC, PMR) regarding Self-Compassion, Emotion Regulation, Psychological Distress, Appraisal, and Rumination. The statistical significance was set to *p* < 0.05 (two-sided). Alpha-error-accumulation was controlled via Bonferroni correction.

## Results

### Descriptive statistics

Participants were on average overweight [BMI: *M* = 28.01, *SD* = 5.93; (105)]. All individuals had a psychiatric diagnosis and 33 (66%) of them at least one comorbid psychiatric diagnosis as reported by the International Classification of Diseases [ICD-10; (106), Chapter F), presented in Table 1. Primarily, people with affective disorders (F30-F39) participated in the study [44 (88%) people; (106)], followed by neurotic, stress-related, and somatoform disorders [F40-F49; (106)]. Thirty-eight (76%) participants took psychotropic drugs, all of them antidepressants, followed by additional neuroleptics intake (14 (36.8%) people). Of note, 24 (48%) participants reported doing sports on a regular basis. Sixteen (32%) people smoked regularly, and four (8%) participants drank alcohol habitually.

**Table 1:** Sample characteristics.

Variable	MSC		PMR		df	*t*/χ^2^	*p*	Cramers *V*/ Cohen’s *d*
	*n_(total)_*	*M* (*SD*) /*f*(%)	*n_(total)_*	*M* (*SD*) /*f*(%)				
Anamnestic data								
Female Sex, *f*(%)	25	17(68)	25	18(72)	1	0.01	0.758	0.04
Age, *M*(*SD*)	25	48.16(13.59)	25	46.28(11.38)	48	0.53	0.598	0.15
Education: ≥ High School, *f*(%)	25	15(60)	25	18(72)	1	0.80	0.370	0.13
In Education/Working, *f*(%)	25	15(60)	25	15(60)	1	0	>0.999	0.00
In Relationship/Married, *f*(%)	25	11(44)	25	11(44)	1	0	>0.999	0.00
Health-related variables								
BMI, *M*(*SD*)	25	27.90(5.40)	24	28.13(6.56)	47	−0.13	0.894	−0.04
Waist-to-hip Ratio, *M*(*SD*)	25	0.87(0.09)	25	0.87(0.09)	48	−0.34	0.732	0.00
Sports, *f*(%)^a^	25	11(44)	25	13(52)	1	0.32	0.571	0.08
Cigarettes, *f*(%)^a^	25	7(28)	25	9(36)	1	0.37	0.544	0.09
Alcohol, *f*(%)^a^	25	2(8)	25	2(8)	1	0	>0.999	0.00
Coffee, *f*(%)^a^	25	20(80)	25	20(80)	1	0	>0.999	0.00
Sugary Drinks, *f*(%)^a^	25	3(12)	25	6(24)	1	1.22	0.269	0.16
(ICD-10) Diagnosis, *M*(*SD*)	25	2.20(1.00)	25	1.88(0.93)	48	1.17	0.247	0.33
F30-F39, *f*(%)	25	23(92)	25	21(84)	1	0.76	0.384	0.12
F40-F49, *f*(%)	25	14(56)	24	12(50)	1	0.18	0.674	0.06
F50-F59, *f*(%)	25	0(0)	25	2(8)	1	2.08	0.149	0.20
F60-F69, *f*(%)	25	3(12)	25	4(16)	1	0.17	0.684	0.06
F90, *f*(%)	25	1(4)	25	1(4)	1	0	>0.999	0.00
Z73.0 Burn Out, *f*(%)	25	5(20)	25	4(16)	1	0.14	0.713	0.05
High BP Family Member, *f*(%)	25	9(36)	25	13(52)	1	1.30	0.254	0.16
High BP, *f*(%)	25	4(16)	25	6(24)	1	0.50	0.480	0.10
Psychotropic Medication, *f*(%)	25	18(72)	25	20(80)	1	0.44	0.508	0.09
Psychotropic Medication, *M*(*SD*)	18	2.44(1.50)	20	2.60(1.50)	36	−0.32	0.752	−0.11
Antidepressants, *f*(%)	18	18(72)	20	20(80)	-	-	-	-
Antiepileptics, *f*(%)	18	2(11.1)	20	5(25)	1	1.22	0.270	0.18
Anxiolytics, *f*(%)	18	2(11.1)	20	1(5)	1	0.49	0.485	0.11
Neuroleptics, *f*(%)	18	7(38.9)	20	7(35)	1	0.06	0.804	0.04
Antipsychotics, *f*(%)	18	2(11.1)	20	2(10)	1	0.12	0.911	0.02
Benzodiazepines, *f*(%)	18	1(5.6)	20	1(5)	1	0.01	0.939	0.01
Hypnotics, *f*(%)	18	0(0)	20	1(5)	1	0.92	0.336	0.16
Cardiovascular medication								
Antihypertensive, *f*(%)	12	3(25)	14	5(35.7)	1	0.35	0.555	0.12
Thyroid, *f*(%)	12	7(58.3)	14	6(42.9)	1	0.62	0.431	0.15
Antihistaminics, *f*(%)	12	2(16.7)	14	0(0)	1	2.53	0.112	0.31
Analgesics, *f*(%)	12	2(16.7)	14	3(21.4)	1	0.09	0.759	0.06
Muscle Relaxants, *f*(%)	12	0(0)	14	1(7.1)	1	0.89	0.345	0.19
Secondary outcome variables								
SCS-D total, *M*(*SD*)	22	3.05(0.82)	22	2.67(0.64)	42	1.71	0.095	0.52
ERQ Reappraisal, *M*(*SD*)	20	4.60(1.33)	22	3.84(1.04)	40	2.08	0.045*^a^	0.64
ERQ Suppression, *M*(*SD*)	21	3.15(1.49)	22	3.43(1.30)	41	−0.65	0.519	−0.20
GSI, *M*(*SD*)	22	2.98(2.47)	22	3.12(2.47)	42	−0.18	0.855	−0.06
Rumination, *M*(*SD*)	24	3.34(1.11)	25	3.26(1.27)	47	0.22	0.824	0.07
DRES-Score, *M*(*SD*)	24	0.74(1.98)	25	0.06(2.44)	47	1.07	0.291	0.31

*M* = Mean; *SD* = Standard Deviation; f = frequency; % = percentage; BMI = Body Mass Index; F30-F39 = Affective Disorders; F40-F49 = Neurotic, Stress-Related, and Somatoform Disorders; F50-F59 = Behavioral Syndromes associated with Physiological Disturbances/Physical Factors; F60-F69 = Personality and Behavioral Disorders; F90 = Attention-Deficit Hyperactivity Disorder; BP = Blood Pressure; SCS-D = Self-Compassion Scale; ERQ = Emotion Regulation Questionnaire; GSI = Global Severity Index (Psychological Distress; BSI-18); DRES-Score = Demand Resource Evaluation Score (Appraisal); ^a^Regular Sport, Cigarettes, Alcohol, Coffee, Sugary Consumption – Participants answered with Yes; Group comparisons were analyzed with chi-square and unpaired t-tests.

* *p* < 0.05.

a ns after Bonferroni correction.

Group comparisons regarding anamnestic data (health-related variables, psychiatric diagnoses, medication) did not reveal significant differences (see Table 1).

Forty-eight participants were included in the HRV and HR-analyzes, 47 for Respiration and 44 for the BP analyzes. If there were no more than 10% missing data in a questionnaire, the mean of the remaining items was inserted for the missing item (107). This procedure was applied for three participants for PA, two for NA and three for SCS-D. For SSP, Appraisal, and Rumination 49 people were enclosed. Data of 44 participants were included in the analyses of SCS-D and BSI-18. For ERQ Reappraisal, 42 people and for ERQ Suppression, 43 people were included (see Tables 1, 2).

**Table 2:** Results of mixed two-way ANOVAs regarding psychophysiological stress response.

Variable	MSC		PMR		ANOVA				
	*n*	*M* (*SD*)	*n*	*M* (*SD*)	Effect	*df*	*F*	*p*	*η_p_^2^*
lnRMSSD (ms)									
Baseline	25	2.73 (0.79)	23	2.79 (0.78)	T	2, 92	1.05	0.353	0.022
Speech-preparation	25	2.80 (0.76)	23	2.77 (0.76)	G	1, 46	<0.01	0.969	<0.001
Speech-recovery	25	2.71 (0.79)	23	2.71 (0.75)	T x G	2, 92	0.47	0.628	0.010
HF FFTlog (ms^2^)									
Baseline	25	4.38 (1.74)	23	4.95 (1.71)	T	2, 92	1.66	0.195	0.035
Speech-preparation	25	4.56 (1.60)	23	4.74 (1.59)	G	1, 46	0.54	0.465	0.012
Speech-recovery	25	4.32 (1.72)	23	4.57 (1.54)	T x G	2, 92	1.13	0.328	0.024
lnSDNN (ms)									
Baseline	25	2.90 (0.70)	23	3.03 (0.65)	T	2, 92	3.29	0.042*^b^	0.067
Speech-preparation	25	3.10 (0.69)	23	3.08 (0.63)	G	1, 46	0.17	0.681	0.004
Speech-recovery	25	2.92 (0.72)	23	3.03 (0.62)	T x G	2, 92	1.18	0.313	0.025
LF FFTlog (ms^2^)									
Baseline	25	4.94 (1.73)	23	5.33 (1.34)	T	2, 92	4.15	0.019*^b^	0.083
Speech-preparation	25	5.47 (1.74)	23	5.57 (1.27)	G	1, 46	0.43	0.518	0.009
Speech-recovery	25	5.07 (1.57)	23	5.38 (1.41)	T x G	2, 92	0.62	0.539	0.013
HR^a^ (bpm)									
Baseline	25	75.04 (12.31)	23	77.58 (13.03)	T	1.75, 80.39	40.93	<0.001***	0.471
Speech-preparation	25	78.67 (11.93)	23	81.56 (11.99)	G	1, 46	0.60	0.444	0.013
Speech-recovery	25	75.22 (12.34)	23	77.94 (12.19)	T x G	1.75, 80.39	0.07	0.911	0.002
SYS BP^a^ (mmHg)									
Baseline	20	117.10 (17.40)	24	116.92 (10.96)	T	1.69, 71.04	25.48	<0.001***	0.378
Speech-preparation	20	125.45 (17.75)	24	124.31 (10.59)	G	1, 42	<0.01	0.981	<0.001
Speech-recovery	20	118.30 (17.11)	24	119.92 (12.74)	T x G	1.69, 71.04	0.75	0.456	0.018
DIA BP^a^ (mmHg)									
Baseline	20	90.18 (10.05)	24	88.29 (9.43)	T	1.63, 68.24	17.63	<0.001***	0.296
Speech-preparation	20	93.45 (9.59)	24	92.67 (9.81)	G	1, 42	0.21	0.650	0.005
Speech-recovery	20	90.65 (10.98)	24	89.33 (9.90)	T x G	1.63, 68.24	0.33	0.678	0.008
PA^a^									
Baseline	25	2.62 (0.66)	25	2.48 (0.71)	T	1.74, 83.55	10.65	<0.001***	0.182
Speech-preparation	25	3.08 (0.81)	25	2.64 (0.89)	G	1, 48	2.15	0.149	0.043
Speech-recovery	25	2.69 (0.84)	25	2.35 (0.91)	T x G	1.74, 83.55	1.72	0.189	0.035
NA^a^									
Baseline	25	1.52 (0.73)	25	1.56 (0.55)	T	1.65, 79.31	11.26	<0.001***	0.190
Speech-preparation	25	1.77 (0.69)	25	1.66 (0.62)	G	1, 48	0.04	0.834	0.001
Speech-recovery	25	1.42 (0.58)	25	1.39 (0.52)	T x G	1.65, 79.31	0.72	0.466	0.015
SSP									
Baseline	24	21.13 (22.76)	25	26.84 (21.91)	T	2, 94	29.60	<0.001***	0.386
Speech-preparation	24	43.71 (26.44)	25	46.20 (29.90)	G	1, 47	0.41	0.527	0.009
Speech-recovery	24	24.71 (19.93)	25	28.64 (28.59)	T x G	2, 94	0.15	0.862	0.003
Respiration^a^ (Hz)									
Baseline	25	0.27 (0.08)	22	0.24 (0.06)	T	1.42, 63.80	10.21	0.001**	0.185
Speech-preparation	25	0.29 (0.06)	22	0.28 (0.05)	G	1, 45	2.32	0.134	0.049
Speech-recovery	25	0.27 (0.09)	22	0.24 (0.07)	T x G	1.42, 63.80	1.24	0.287	0.027

ln = Natural Logarithmic Normalization of the Data; lnRMSSD = Logarithmized Root Mean Square of Successive Differences; lnSDNN = Logarithmized Standard Deviation of NN Intervals; HF = High-Frequency Band; LF = Low-Frequency Band; HR = Heart Rate; SYS BP = Systolic Blood Pressure; DIA BP = Diastolic Blood Pressure; ms = milliseconds; ms^2^ = milliseconds squared; FFTlog = logtransformed with Fast Fourier Transformation; bpm = beats per minute; mmHg = millimetres of mercury; PA = Positive Affect, PA activation = Facet of PA; NA = Negative Affect; SSP = Subjective Stress Perception; Hz = Hertz; time (baseline, speech prep., recovery); MSC = Mindful Self-Compassion; PMR = Progressive Muscle Relaxation; G = group; T = time.

* *p* < 0.05.

** *p* < 0.01.

*** *p* < 0.001.

a Greenhouse–Geisser correction.

b ns after Bonferroni correction.

### HRV-norms and respiration

Published resting short-term HRV norms of approximately 5 min length collected in 21,438 non-clinical individuals in sitting or lupine position (74) were compared to the 3 min baseline measurement in this clinical sample for exploratory reasons. The present sample showed significantly lower RMSSD, HF-HRV, and SDNN during baseline measurement as compared to the non-clinical norms. For LF-HRV, no significant difference was found (see Table 3). According to Shaffer and Ginsberg (74) participants should breathe at 11–20 breaths per minute (BPM) that short-term HRV values are adequate. Respiration rate for baseline measurement was on average 15 BPM (*M* = 15.30, *SD* = 4.46), for speech preparation 17 BPM (*M* = 17.21, *SD* = 3.12), and for recovery 15 BPM (*M* = 15.41, *SD* = 4.86). Mixed ANOVA with Respiration rate showed no significant main difference between MSC and PMR group (see Table 2). Therefore, no further analyzes including Respiration deemed necessary.

**Table 3:** Non-clinical short-term HRV norms (*n* = 21,438) in comparison to clinical sample (*n* = 48) (108).

	Non-clinical		Clinical		*t*(21484)	*p*	Cohen’s *d*
	*M*	*SD*	*M*	*SD*			
RMSSD (ms)	42	15	20.54	14.05	9.90	<0.001***	1.431
SDNN (ms)	50	16	23.81	14.80	11.33	<0.001***	1.637
HF (ms^2^)	657	777	312.98	416.78	3.07	0.002**	0.444
LF (ms^2^)	519	291	467.22	726.23	1.22	0.221	0.176

RMSSD = Root Mean Square of Successive Differences; SDNN = Standard Deviation of NN Intervals; HF ms^2^ = Absolute Power of the High-Frequency Band; LF ms^2^ = Absolute Power of the Low-Frequency Band; M = Mean; SD = Standard Deviation. Adapted from Shaffer, F., & Ginsberg, J. P. (2017). An overview of heart rate variability metrics and norms. Frontiers in public health, 5, 258. https://doi.org/10.3389/fpubh.2017.00258.

** *p* < 0.01.

*** *p* < 0.001.

### Primary outcome results

For all Mixed ANOVA calculations, prerequisites have been checked. When the assumption of sphericity was not fulfilled, the Greenhouse–Geisser correction was applied (see Table 2^a^) (109). Normal distribution was assessed by Shapiro–Wilk test (*p* > 0.05), and if violated, ANOVAs were still calculated due to the central limit theorem (110). Box’s Test showed homogeneity of covariances for all calculations [*p* > 0.05; (111)]. Except for SSP in recovery phase, homogeneity of variances was given by Levene’s test (*p* > 0.05). Post-hoc comparisons were still interpreted for SSP (112).

#### Stress induction: changes in HRV, HR, and BP

For HRV, HR, and BP, calculations revealed significant main effects of time for SDNN (*p* = 0.42; *ns* after Bonferroni correction), LF (*p* = 0.019; *ns* after Bonferroni correction), HR, SYS BP, and DIA BP (all *p* < 0.001), respectively. There were no significant main effects group or interaction effects regarding HRV, HR and BP (see Table 2).

For SDNN, pairwise comparison showed no significant results between baseline and speech preparation (*p* = 0.106), and speech preparation and recovery (*p* = 0.130), respectively. LF-HRV was significantly lower during baseline (*M* = 5.13, *SD* = 1.55) than during speech preparation (*M* = 5.52, *SD* = 1.52, *p* = 0.023). No significant changes were found towards recovery (*p* = 0.151). For HR, pairwise comparisons indicated a significant increase from baseline (*M* = 76.26, *SD* = 12.59) to speech preparation (*M* = 80.06, *SD* = 11.92, *p* < 0.001) and decrease from speech preparation to recovery (*M* = 76.52, *SD* = 12.22, *p* < 0.001). Pairwise comparison for SYS BP revealed a significant rise from baseline (*M* = 117.00, *SD* = 14.07) to speech preparation (*M* = 124.83, *SD* = 14.13, *p* < 0.001). No significant decrease towards recovery was found (*p* = 0.059). DIA BP revealed a significant increase from baseline (*M* = 89.15, *SD* = 9.65) to speech preparation (*M* = 93.02, *SD* = 9.61, *p* < 0.001) and a decline from speech preparation to recovery (*M* = 89.93, *SD* = 10.31, *p* < 0.001).

#### Stress induction: changes in PA, NA, SSP

As detailed in Table 2 the results of the stress intervention indicated expected reactions with significant main effects time on PA, NA and SSP. PA showed a significant rise from baseline (*M* = 2.55, *SD* = 0.68) to speech preparation (*M* = 2.86, *SD* = 0.87, *p* < 0.001) and significant decline to recovery phase (*M* = 2.52, *SD* = 0.88, *p* = 0.003).

For NA, there was no significant increase to speech preparation (*M* = 1.72, *SD* = 0.65, *p* = 0.085), but a significant decrease to recovery (*M* = 1.41, *SD* = 0.55, *p* < 0.001). SSP was significantly higher at speech preparation (*M* = 44.98, *SD* = 27.99) than at baseline (*M* = 24.04, *SD* = 22.29, *p* < 0.001) and lower at recovery (*M* = 26.71, *SD* = 24.55, *p* < 0.001) than at speech preparation. No significant main effects group nor interaction effects were found (see Table 2).

### Secondary outcome results

For unpaired *t*-tests, normal distribution was given, as assessed by Shapiro–Wilk test (*p* > 0.05) except for PMR group regarding Psychological Distress and Rumination (110). Homoscedasticity was evident in all calculations (Levene’s test: *p* > 0.05). For Self-Compassion, Suppression, Psychological Distress, Appraisal, and Rumination, no group differences were detected. The MSC group showed significantly higher values in Reappraisal than the PMR group (see Table 1), indicating a medium effect size (Cohen’s *d* = 0.64; *ns* after Bonferroni correction). However, this result was non-significant after Bonferroni correction.

## Discussion

The main purpose of this study was to determine whether the psychophysiological stress response differed in inpatients attending the MSC or PMR program at the end of their 6-week psychiatric rehabilitation stay in the Sonnenpark Neusiedlersee clinic. Overall, no difference was found. Participants were comparable on several anamnestic variables such as diagnoses or medication intake, Self-Compassion, Suppression, and Psychological Distress, as well as in HRV, HR, BP, PA, NA, SSP, Respiration rate, Appraisal, and Rumination in the course of the stress induction. While participants in the MSC group exhibited increased Cognitive Reappraisal as an emotion regulation strategy, as compared to the PMR group, this difference did not remain significant after controlling for multiple testing.

On a psychophysiological level, substantial stress-related changes were observed in both groups, thus demonstrating effectiveness of the stress task. In particular, alterations in SDNN, LF-HRV, HR, BP, PA, NA, and SSP throughout the three measurement points (baseline, speech prep., recovery) of the stress task could be observed. However, no significant changes were detected for RMSSD and HF-HRV.

This study is, to the authors’ knowledge, the first to compare MSC training with an active PMR control group regarding psychophysiological stress reactivity. Initial indications for group differences were provided by the study of Gaiswinkler et al. (51), where MSC training was associated with higher self-compassion and happiness than PMR training after a 6-week intervention in the Sonnenpark Neusiedlersee clinic. In this study, a tendency for higher Self-Compassion in the MSC group (*p* = 0.095) was found, as well as a superior Cognitive Reappraisal (*p* = 0.045), which confirms recent findings on how self-compassion increases the ability of cognitive reappraisal as self-compassion helps us to look at ourselves and situations we are in with more kindness (47, 113, 114). Higher reappraisal could also help to better deal with stressful situations as they are perceived as evolving (115, 116). Besides, the MSC and the PMR training seem to produce quite similar results like several other studies comparing mindfulness and PMR (66, 68, 117, 118). Although - resonating with previous research - our findings hint towards effects of MBIs on psychological stress response regarding SSP (119), Emotion Suppression (120), Psychological Distress (117), Appraisal (85, 121), and Rumination (49, 122), this study did not employ a placebo group which significantly restricts the generalizability of our findings.

In psychophysiological research, heterogeneous findings exist where differences between mindfulness and relaxation interventions were found (53, 119, 123). Especially for brief self-compassion and meditation interventions on physiological stress reactivity, adaptive psychophysiological reactions could not be confirmed (32, 124). These results may be due to short-term interventions that have predominantly been conducted in healthy or subclinical populations (53, 119). In contrast, in this study, a 6-week-long intervention was performed in a clinical population. Generally speaking, there are studies showing mindfulness and self-compassion training increasing HRV and buffering psychophysiological stress response (33, 55, 57, 58, 125, 126). Similar to self-compassion programs, PMR may buffer psychophysiological stress reactivity (67, 127). These studies suggest that both programs might be equivalent. To further test this hypothesis future studies should apply equivalence testing on the effects of both relaxation techniques.

A moderate stress response was evident in individual psychophysiological parameter. The stress response of HR, BP, NA, and SSP was observed congruent with literature since both groups showed a rise from baseline to speech preparation and a decline towards recovery (82, 119, 125, 128, 129). Additionally, a significant increase to speech preparation was observed in PA, which seems surprising at first glance (130, 131). Nevertheless, no parasympathetic change regarding RMSSD and HF-HRV was observed, possibly indicating no adaptive stress behavior (20, 132). This is rather uncommon in stress experiments, but could be due to the clinical population and their low flexibility of the ANS (18, 19, 133, 134).

While we did not find significant differences between MSC and PMR, future investigations might examine characteristics of specific groups for whom MSC works particularly well or people who do not benefit from it. E.g. previous research identified neuroticism and conscientiousness as possible moderators of mindfulness based interventions (135). Regarding the moderate stress response, the stress induction might be applicable for future stress experiments. Especially in clinical populations, it may be advantageous not to obtain a strong but moderate stress response due to vulnerability.

### Limitations and future research

The first point to be mentioned is the sample size in this study. This only allows for large effects to be revealed by statistical evaluation (136, 137). To detect medium and small effects, a larger sample size is certainly required. Additionally, a within subject design study would be desirable (73). Quintana and Heathers (131) recommend collecting multiple data from one individual at several times in psychophysiological studies. Due to the selected stressor, we were not able to apply the speaking task twice, otherwise a learning effect and therefore an inadequate stress response might have been detected (73). For future studies, however, we should consider presenting a different stress task at the beginning of the 6-week stay as well as at the end to reveal the changes within a person pre- and post-treatment (73). Recently, Asbrand et al. (138) showed that the standardized TSST could be used in repeated measures, which might constitute an additional approach for future replication studies.

Besides, a non-clinical control group additionally to the active PMR control group would be essential to compare clinical and non-clinical subjects (139). In this study, an attempt was made to compare HRV norm values in non-clinical studies with the present sample, which indicated lower HRV values in comparison to norms. However, to our knowledge, no norm values exist for stress reactivity (74). Still, studies show blunted HRV reactivity in a stress experiment in patients with mental illness as compared to healthy individuals, which is in line with the Neurovisceral Integration Model and makes comparative values preferable in future studies (18, 19, 21, 79, 140). Considering that affective disorders are most frequent in the rehabilitation clinic, this is of particular interest since Jandackova et al. (141) consider HRV as an influencing factor on the onset of depression.

In the present study, the two instructors, teaching MSC and PMR, were not supervised. Thus, it would be interesting to address the training of the instructors as well as their mindset. Showing compassion for others requires us to be aware of our own pain and may help clinicians be more effective in therapy (142).

## Conclusion

In sum, this study was the first one to compare MSC and PMR program with respect to psychophysiological stress reactivity with clinical context. The results of this experiment showed no significant difference in the psychophysiological stress responses of inpatients at the end of their psychiatric rehabilitation stay, thus suggesting no difference in the response profile of both the MSC and PMR program.

Yet, larger studies will be needed to further explore differences and similarities of both interventions in more detail.

## Data availability statement

The original contributions presented in the study are included in the article/supplementary material, further inquiries can be directed to the corresponding author.

## Ethics statement

The studies involving human participants were reviewed and approved by Ethics Committee of the University of Graz. The patients/participants provided their written informed consent to participate in this study.

## Author contributions

SK, AA, PK, EP, AS, and H-FU conceptualized the study. SK, CS, and AA collected the data. SK and CT analyzed the data. SK interpreted the data. SK, JF, AA, AS, and H-FU drafted the manuscript. AS, CT, CR, JF, AA, and H-FU critically reviewed it. All authors contributed to the article and approved the submitted version.

## Conflict of interest

The authors declare that the research was conducted in the absence of any commercial or financial relationships that could be construed as a potential conflict of interest.

## Publisher’s note

All claims expressed in this article are solely those of the authors and do not necessarily represent those of their affiliated organizations, or those of the publisher, the editors and the reviewers. Any product that may be evaluated in this article, or claim that may be made by its manufacturer, is not guaranteed or endorsed by the publisher.
